# Functions of the bone morphogenetic protein signaling pathway through non-coding RNAs

**DOI:** 10.1016/j.ncrna.2022.07.002

**Published:** 2022-07-09

**Authors:** Ural Mukhametov, Sergey Lyulin, Dmitry Borzunov, Galina Sufianova, Alina Shumadalova, Daming Zhang, Ilgiz Gareev

**Affiliations:** a Republican Clinical Hospital. G.G. Kuvatova, Ufa, 450071, Russian Federation; b Carmel Medical Center, Chelyabinsk, 454080, Russian Federation; c Ural State Medical University, Ekaterinburg, 620028, Russian Federation; d Рeoples’ Friendship University of Russia (RUDN University), 6 Miklukho-Maklaya Street, Moscow, 117198, Russian Federation; e Bashkir State Medical University, Ufa, Russian Federation; f Department of Pharmacology, Tyumen State Medical University, Tyumen, Russia; g Department of Neurosurgery, The First Affiliated Hospital of Harbin Medical University, Harbin, China

**Keywords:** Bone morphogenetic proteins, miRNAs, lncRNAs, circRNAs, Oncogenesis, Osteoporosis, Biological processes

## Abstract

Bone morphogenetic proteins (BMPs) are proteins of the transforming growth factor-β (TGF-β) family, which plays an important role in the formation of skeletal and cartilage tissue and their regeneration. BMPs play a key role in the formation of new blood vessels and promote the migration, proliferation, and differentiation of mesenchymal stem cells (MSCs) into chondroblasts and osteoblasts. It is known that malfunction of BMPs signaling can cause a disease state. Epigenetic regulation of expression plays a key role in the control of many cellular processes. Important participants in this regulation are non-coding RNAs (ncRNAs), which are RNA molecules that are not translated into proteins. The best known of these are microRNAs (miRNAs), long non-coding RNAs (lncRNAs), and circular RNAs (circRNAs). In addition, the results of many studies make it possible to establish an unambiguous functional relationship between these ncRNAs. Being involved in the regulation of a large number of target genes responsible for the life of the cell, miRNAs, lncRNAs, and circRNAs are essential for the normal development and functioning of the body, and the violation of their functions accompanies the development of many pathophysiological processes including oncogenesis. In the present review, we discuss different insights into the regulation of BMPs signaling pathway by miRNAs, lncRNAs and circRNAs governed.

## 1 Introduction

Bone morphogenetic protein (BMPs) are a group of 20 types of pleiotropic ligand proteins that interact with specific receptors, co-receptors, and signaling-modulating proteins of the transforming growth factor-β (TGF-β) superfamily. BMPs stimulate the formation of new blood vessels and promote migration, proliferation, and differentiation of mesenchymal stem cells (MSCs) into chondroblasts and osteoblasts. BMP-2, -4, -6, -7 and -9 have the most pronounced osteogenic properties [1,2].

BMPs activity is mediated by the activation of downstream extracellular signaling pathways, which in turn are initiated by BMPs binding to transmembrane receptor protein serine/threonine kinases (RSTK). This interaction triggers specific intracellular signaling pathways that control the transcription of specific target genes [3]. There are two types of BMPs receptors on the cell membrane. The activin type I receptors (Act-RI), including Act-RIA and Act-RIB, and activin type II receptors (Act-RII), including Act-RIIA and Act-RIIB [1,2]. Both types of receptors are indispensable for the formation of a functional complex that initiates subsequent signaling cascades. By binding to various receptor complexes, BMPs activates two major downstream signaling pathways: Smad-dependent and Smad-independent. Activated BMPs receptors phosphorylate the transcription factors Smad1, Smad5, and Smad8, which in the cytoplasm of the cell assemble with Smad4 into a complex with subsequent movement to the nucleus, where they regulate the transcription of target genes, such as inhibitor of differentiation/DNA binding 1 (ID1), distal-less homeobox 5, runt-related transcription factor 2 (Runx2) and osterix [4]. At the same time, BMPs activates a number of Smad-independent downstream signaling pathways, including mitogen-activated protein kinase pathways such as p38, c-Jun N-terminal kinase, and extracellular signaling kinase pathways [5]. They play an important role in BMPs-induced osteogenic events. During osteoblastogenesis, MSCs proliferate and synthesize alkaline phosphatase (ALP) and osteocalcin, which leads to mineralization and bone formation [6]. Recent findings have established that the levels of BMPs are modulated by non-coding RNAs, including miRNAs, lncRNAs or circRNAs [7,8].

Non-coding RNAs (ncRNAs) are a class of RNA molecules that are not translated into protein. Based on their length, ncRNAs can be divided into three categories: 1) ncRNAs longer than 200 nucleotides (nt), including long non-coding RNAs (lncRNAs); 2) ncRNAs shorter than 200 nt but longer than 40 nt, such as transfer RNA (tRNA) and small nucleolar RNA (snoRNA); and 3) ncRNA short than 40 nt like microRNAs (miRNAs) [9]. Unlike linear ncRNAs mentioned above, circular RNAs (circRNAs) is a newly defined type of ncRNA that forms a covalently closed loop, with no 5′ and 3′ polar [10]. In general, ncRNAs are found to participate in multiple biological processes, regulate physiological processes and the processes of development of various human diseases. NcRNAs can interact with and regulate different kinds of molecules through different molecular mechanisms, creating a complex network [9,11]. NcRNA-mediated networks not only contain interactions between RNAs, but are also involved in genomics and proteomics in both the nucleus and cytoplasm. In the present review, we discuss and summarize BMPs signaling pathway regulation that are mediated by miRNAs, lncRNAs and circRNAs in essential biological processes involving angiogenesis, osteogenesis or neurogenesis and in pathological processes such as oncogenesis and osteoporosis.

## 2 MiRNAs and BMPs signaling pathway

MiRNAs are short, averaging 18–22 nucleotides, single-stranded nсRNAs that regulate gene expression at the post-transcriptional level by binding to the 3′-untranslated region (3′-UTR) of specific messenger RNA (mRNA) targets, resulting in a decrease in protein expression by blocking translation and (or) contributing to the degradation of mRNA targets [10,11]. There are many studies proving the importance of miRNAs in the regulation of many biological processes, such as neurogenesis, cell proliferation and differentiation, apoptosis, regulation of immune processes, fat metabolism, glucose homeostasis, etc. [12]. It has been shown that dysregulation or aberrant miRNA expression associated with these processes leads to the development of various human diseases, such as oncology, cardiovascular, autoimmune, and neurodegenerative diseases [12].

BMPs signaling pathway cascades are post-transcriptionally modulated by different miRNAs that stimulate or inhibit osteogenesis, angiogenesis or neurogenesis. Specifically, several miRNAs can directly increase or reduce the expression of the genes coding for components of the BMPs signaling pathways or the transcription factors involved in cells differentiation, ultimately exerting both stimulatory and inhibitory effects on osteogenesis, angiogenesis or neurogenesis [13,14].

Osteoblast differentiation is a key step in skeletal development. Activation of the TGF-β and BMPs signaling pathways is involved in the differentiation of MSCs into the osteogenic lineage. For example, BMP-2 and BMP-7 act as potential differentiators through Smad-mediated activation of major osteoblast target genes such as Runx2 [5,15]. A number of miRNAs that are modulated BMPs signaling have recently been reported to regulate osteoblast differentiation both positively and negatively. MiR-20a is one of such miRNAs. Antagonists of the BMPs signaling pathway, namely the activin membrane-bound inhibitor homolog (BAMBI) and cysteine-rich motor neuron 1 protein (Crim1), are targets of miR-20a [16]. BAMBI is a pseudo-receptor whose structure is close to type I transmembrane receptors that can bind to BMPs; because it lacks an intracellular kinase domain, message induction is abolished, resulting in inhibition of the BMPs signaling pathway [17]. Crim1 is another transmembrane protein often present in cell membranes. Crim1 can interact with BMPs and disrupt its synthesis [18]. Peroxisome proliferator-activated receptor γ (PPARγ) is an alternative target for miR-20a, which belongs to the nuclear receptor family [19]. Although PPARγ is a key factor in adipocyte differentiation, PPARγ has a negative regulatory effect on osteoblast differentiation [19]. Activation of PPARγ is accompanied by a decrease in the expression level of mRNA and Runx2 protein. In addition, PPARγ strongly interacts with Runx2, preventing its transcriptional activity [20]. Another PPARγ pathway through which it suppresses osteoblast differentiation is through inhibition of BMP-2 expression [21]. Activation of miR-20a results in an increase in Runx2 expression and activation of the BMPs signaling pathway through inhibition of PPARγ [19]. It has been shown that during aging, the expression level of PPARγ increases, which leads to increased binding of MSCs to adipocytes, so the role of PPARγ in the development of osteoporosis with aging is the subject of study [22]. In addition, the expression level of miR-20a in MSCs from young donors is higher compared to adults, and the expression level of miR-20a decreases with human aging [16]. Taken together, these data highlight the putative role of miR-20a in osteoporosis.

It is known that inactivation of the BMPs signaling pathway leads to the development of various vascular disorders [23,24]. For example, cells isolated from patients with heritable pulmonary arterial hypertension (HPAH) show mutations in the type 2 bone morphogenetic protein receptor (BMPR2) or Smad9 [25]. It should be noted that, although the loss of Smad9 function in the canonical BMPs signaling pathway is largely compensated by Smad1 and Smad5, the Smad9 mutation itself is completely abolished by miRNA induction [26]. This result suggests that miRNA regulation by BMPs signaling is involved in normal vascular development and homeostasis. During vascular development, BMP signaling increases the expression of genes specific to vascular smooth muscle cells (VSMCs) and inhibits cell proliferation and migration, leading to differentiation of VSMCs [27]. Several miRNAs have been found to be regulated by BMPs signaling where they are responsible for VSMCs differentiation and proliferation under physiological or pathological conditions. There is ample evidence that the miR-143/-145 cluster is involved in the regulation of differentiation and proliferation of VSMCs [28]. BMPs signals activate transcription of the miR-143/145 cluster genes through a consensus sequence called the CArG box by serum response factor (SRF) and myocardin related transcription factor A (MRTFA) [28].

In addition to their well-characterized role in bone development, BMPs are critical to neurodevelopment [29]. Mao et al. in their study studied the change in the level of miRNA expression in the cerebral cortex of mice at different stages of their development [30]. It has been shown that miR-17 expression was downregulated in the developing cortex. Since miR-17 downregulates BMPR2 expression, downregulation of miR-17 expression activates the BMPs signaling pathway, which facilitates astrocytogenesis during cell differentiation. However, miR-17 promotes neural progenitor cell proliferation, and inhibition of BMPs signaling promotes miR-17-mediated increase in neural progenitor cell proliferation. Therefore, miR-17 plays an important role during cortical development by modulating the BMPs signaling pathway. More illustrative examples of miRNAs functioning by activating BMPs signaling pathways are presented in Table 1 [[31], [32], [33], [34], [35], [36], [37], [38], [39], [40]].

**Table 1:** Functions of the bone morphogenetic protein (BMP) signaling pathway through miRNAs.

miRNA	BMP signaling pathway	Biological process	Targets	Function	References
miR-30b/c	BMP-2 and Smad-independent pathway	Vascular	Runx2	Promote of VSMCs calcification	31
miR-302	BMP-4, Smad4 and BMPRII	Vascular	HDAC	Inhibit of the contractile phenotype of VSMCs	32
miR-96	BMP-4	Vascular	Trb3	Promote of the contractile phenotype of VSMCs	33
miR-133 and miR-135	BMP-2 and Smad5	Osteogenesis	Runx2	Inhibit the differentiation of osteoprogenitors	34
miR-141 and miR-200a	BMP-2	Osteogenesis	Dlx5	Suppression of ALP activity, modulate the BMP-2-stimulated pre-osteoblast differentiation	35
miR-208 and miR-370	BMP-2	Osteogenesis	Ets1	Suppression of ALP and mineralization	36, 37
miR-27a	BMP-2, BMPR1A and Smad9	Osteogenesis	Satb2, Runx2 and ATF4	Promote osteoblast differentiation	38
miR-22	BMP-2	Neurogenesis	N-myc	Anti-proliferative effect, significantly increasing the cell cycle duration in cerebellar granular neuronal precursors	39
miR-134	BMP-7	Neurogenesis	Chrdl-1	Modulates dendritic maturation	40

**Abbreviations:** BMP-2, bone morphogenetic protein 2; BMP-7, bone morphogenetic protein 7; BMP-4, bone morphogenetic protein 4; BMPR1a, bone morphogenetic protein receptor, type IA; VSMCs, vascular smooth muscle cells; ALP, alkaline phosphatase; Runx2, runt-related transcription factor 2; HDAC, histone deacetylase; Trb3, tribbles-like protein 3; Dlx5, distal-less homeobox 5; Ets1, V-ets erythroblastosis virus E26 oncogene homolog 1; Satb2, AT-rich sequence-binding protein 2; ATF4, activating transcription factor 4; Chrdl-1, chordin-like 1.

## 3 LncRNAs and BMPs signaling pathway

LncRNAs are non-coding transcripts over 200 nucleotides long, which are regulators of both physiological intracellular processes and intercellular interactions, and the pathogenesis of various human diseases. LncRNAs have been shown to play a critical role in transcription, imprinting and DNA methylation, translation, chromatin modification, and cell cycle regulation [10,11]. LncRNAs play an important role in the oncogenesis, especially in the development and progression of tumors through the wingless‐type (Wnt), Hedgehog, Notch, and phosphoinositide 3-kinases (PI3Ks)/protein kinase B alpha (AKT)/mammalian target of rapamycin (mTOR) signaling pathways [41]. Dysregulation of several lncRNAs has been found in various cancer types such as breast, ovarian, cervical, and prostate cancers, suggesting the use of lncRNAs as markers for cancer detection and prognosis, as well as for therapeutic purposes [41,42].

LncRNAs are known to function as competing endogenous RNAs, binding to miRNAs responses to effectively sequester miRNAs, thereby regulating biological processes such as osteogenesis [43]. This feature of lncRNAs effectively prevents miRNAs from binding to their target mRNA molecules, the translation of which they are usually able to suppress in a highly specific manner by binding to their 3′-UTR. The lncRNA/miRNA/mRNA axis has been reported to exist widely in various disease contexts (e.g. tumors), but there is limited research on how this axis functions in the context of osteogenic differentiation, making the study of these functions and their underlying molecular mechanisms an important research area [43,44]. Li et al. found that BMP-9 stimulation could expedite peak expression of HOX transcript antisense RNA (HOTAIR) [45]. Moreover, silencing of HOTAIR could promote cell proliferation and inhibit BMP-9-induced differentiation of MSCs both *in vivo* and *in vitro*. The authors also found that inactivation HOTAIR can to inhibit bone generation and downregulate Runx-2 as well as osteocalcin (OCN), which could theoretically down-regulate the expression of osteopontin (OPN) and thus lead to further inactivation of HOTAIR. Thus, it is conceivable that OPN and HOTAIR could form a positive feedback loop in the regulation of MSCs osteogenesis. In other study, Su et al. demonstrated that HOTAIR regulates fracture healing in osteoporotic rats through inhibition on miR-17-5p [46]. HOTAIR is also known to be involved in ubiquitin-dependent protein degradation, as evidenced by its association with E3 ubiquitin ligases and carrier RNA-binding domains such as Dzip3 and Mex3b [47]. The discovery that Mex3b can help HOTAIR interact with Smad4, a key component of BMPs signaling pathways, strongly points to a role for HOTAIR in regulating TGF-β superfamily signaling.

In their study, Fu et al. showed that lncRNA PVT1 was highly expressed in human glioma tissues and cell lines [48]. It has been proven that there is a regulatory relationship between lncRNA PVT1 and miR-128-3p, as well as between miR-128-3p and GREM1. In addition, miR-128-3p expression was suppressed by GREM1 activation in glioma tissues compared to neighboring normal tissues. Thus, the authors showed that GREM1 overexpression promotes proliferation and metastasis of glioma cells, while the use of miR-128-3p mimic inhibits the biological functions of glioma cells by targeting the GREM1 3′-UTR. Ultimately, they demonstrated that the lncRNA PVT1 acts as a miR-128-3p sponge and influences the regulation of the BMPs signaling pathway as well as the downstream signaling proteins BMP-2 and BMP-4 through the regulation of GREM1. More illustrative examples of the lncRNA/miRNA/mRNA axis functioning by activating BMPs signaling pathways are presented in Table 2 [[49], [50], [51], [52], [53], [54], [55], [56], [57], [58]].

**Table 2:** Functions of the bone morphogenetic protein (BMP) signaling pathway through lncRNAs.

lncRNA	BMP signaling pathway	Biological or pathological process	Targets	Function	References
MALAT1	BMP-2	Vascular and osteogenesis	miR-494/TLR2/SP1 axis	MALAT1 as a crucial regulator of angiogenesis and osteogenic differentiation	49
ANCR	BMP-2	Vascular	Runx2	Inhibit the osteoblastic differentiation of VSMCs and attenuate arterial calcification through activating autophagy *in vivo*	50
MALAT1	BMP-7	Vascular	miR142-3p/PDGF/ATG7	Promotes the transformation of VSMCs from contraction to synthetic phenotypes.	51
Lnc-OAD	BMP-2	Osteogenesis	AKT/Osterix axis	Induces osteoblast differentiation	52
TUG1	BMP-2	Osteogenesis	miR-214	Knockdown of TUG1 represses viability, migration and differentiation and induces apoptosis of osteoblasts	53
LINC01535	BMP-2	Osteogenesis	miR-3619-5p	Promote osteogenic differentiation	54
MALAT1	BMP-2 and Smad1	Oncogenesis	miR-26a-5p	Inhibit of human colorectal cancer cells proliferation and migration partially	55
FGD5-AS1	Smad6	Oncogenesis	miR-196a-5p	Inhibits migration gastric cancer cells, cell viability, and EMT transformation rate	56
MEG3	BMP-4, p-Smad1/5/8, and Smad1	Osteoporosis	DEPTOR and Runx2	Understanding of the regulatory mechanisms of the MEG3 by BMP signaling pathways, and might contribute to bone tissue engineering and the treatment of osteoporotic diseases.	57
MSC-AS1	BMP-2 and p-Smad1/5/8	Osteoporosis	miR-140-5p/Runx2/Osterix	Promote the osteogenic differentiation of BMSCs, thus alleviating the progression of osteoporosis	58

**Abbreviations:** BMP-2, bone morphogenetic protein 2; BMP-4, bone morphogenetic protein 4; BMP-7, bone morphogenetic protein 7; MALAT1, metastasis associated lung adenocarcinoma transcript 1; ANCR, lncRNA anti-differentiation non-coding RNA; Lnc-OAD, lncRNA associated with osteoblast and adipocyte differentiation, transcribed from 1700018A04Rik gene; TUG1, taurine upregulated gene 1; LINC01535, long intergenic non-protein coding RNA 1535; FGD5-AS1, FGD5 antisense RNA 1; MEG3, maternally expressed 3; MSC-AS1, MSC antisense RNA 1; TLR2, Toll-like receptor 2; SP1, specificity protein 1; Runx2, runt-related transcription factor 2; VSMCs, vascular smooth muscle cells; DEPTOR, DEP domain containing mTOR interacting protein; PDGF, platelet-derived growth factor; ATG7, autophagy related 7; EMT, epithelial–mesenchymal transition; BMSCs, bone marrow stromal cells.

## 4 CircRNAs and BMP signaling pathway

СircRNAs are type of single-stranded RNAs that, unlike linear RNA, forms a covalently closed continuous loop. In circRNA, the 3′ and 5′ ends normally present in an RNA molecule are joined together [10]. This feature gives circRNA numerous properties, many of which have only recently been identified. Many types of circRNA arise from genes that code for protein differently. Some circRNAs have been shown to encode proteins. Several types of circRNA have recently shown potential as gene regulators [59,60]. The biological function of most cirсRNAs is unclear. Nevertheless, it is known that circRNAs participate in various cellular processes, including cell proliferation, apoptosis, angiogenesis, and regulate osteogenic differentiation [10,61]. Because circRNAs do not have 5′ or 3′ ends, they are resistant to exonuclease-mediated degradation and presumably more stable than most linear RNAs in cells. CircRNAs has been linked to some diseases such as cancers and osteoporosis [62,63].

Osteoporosis in its genesis is a multifactorial disease, in the formation of which a significant contribution is made by factors involved in the processes of bone remodeling and being target molecules for the search for new drugs [64]. These include molecules of the cytokine tumor necrosis factor ligand superfamily member 11 (RANKL)/RANK/osteoprotegerin (OPG) axis, Wnt/B-catenin signaling pathway, proteins of the TGF-β family — BMPs and activin, as well as a number of proteins that exhibit the properties of agonists or antagonists of these target molecules [65]. It has been reported that circRNAs have rich miRNA binding sites where they are able to play the role of competing endogenous RNA, acting as miRNAs sponges in cells, and are able to regulate the pathogenesis of some diseases [62,63]. This network abolishes the regulatory and inhibitory effects of miRNAs on their target genes and thus increases the level of expression of target genes. It is generally accepted that the circRNA-miRNA network is closely associated with osteogenic differentiation [61]. In a recent study, Zhou et al. demonstrated that circRNA circ_0000020 promotes osteogenic differentiation to reduce osteoporosis via sponging miR-142-5p to up-regulate BMP-2 [66]. In this study, the authors indicated that circ_0000020 silencing inhibits bone marrow mesenchymal stem cells (BMSCs) osteogenic differentiation. This result to describe the regulatory role of circ_0000020/miR-142-5p/BMP-2 axis in BMSCs osteogenic differentiation, thus providing new theoretical basis for developing new drug targets in osteoporosis.

To date, the mechanism of circRNAs in postmenopausal osteoporosis remains largely unknown. However, there are a number of evidence-based studies on the role of circRNAs in the pathogenesis of postmenopausal osteoporosis. For instance, Qiao et al. elucidated that circRNA_0048211 was downregulated in human BMSCs were collected from progression of postmenopausal osteoporosis patients, which was gradually enhanced during osteogenesis [67]. Overexpression of circRNA_0048211 markedly upregulated BMP-2, ALP activity, and mineralization ability in human BMSCs, suggesting the involvement of circRNA_0048211 in osteogenesis. CircRNA_0048211/miRNA-93-5p/BMP-2 axis provides new targets for prevention and treatment of postmenopausal osteoporosis. Thus, we seek to explore which circRNAs are involved in the critical pathways of some biological (e.g. osteogenesis) and pathological process (e.g. osteoporosis) by regulatory BMPs signal pathways (Table 3) [[68], [69], [70], [71], [72]].

**Table 3:** Functions of the bone morphogenetic protein (BMP) signaling pathway through circRNAs.

circRNA	BMP signaling pathway	Biological or pathological process	Targets	Function	References
mm9_circ_009056	BMP-7	Osteogenesis	CGRP, Runx2 and miR-22-3p	Promote osteogenesis and cell proliferation	68
circ_0129047	BMPR2	Oncogenesis	miR-1206	Tumor suppressive role in lung adenocarcinoma progression	69
has_circ_0003506	BMPR2	Oncogenesis	miR-1256	Knockdown of circ_0003506 suppressed radioresistance in gastric cancer	70
circ_0007059	BMP-2	Osteoporosis	miR-378	Promotes the osteoclastogenesis of hBMSCs	71
hsa_circ_0005752	BMP-2	Osteoporosis	miR-496/MDM2-p53, Runx2, ALP, Osterix, and OCN	Promotes osteogenic differentiation, as shown by enhancing ALP and ARS staining intensity.	72

**Abbreviations:** BMP-2, bone morphogenetic protein 2; BMP-7, bone morphogenetic protein 7; BMPR2, bone morphogenetic protein receptor type 2; CGRP, calcitonin gene-related peptide; Runx2, runt-related transcription factor 2; ALP, alkaline phosphatase; ARS, aminoacyl-tRNA synthetase; hBMSCs, human bone marrow stromal cells; MDM2, mouse double minute 2 homolog; OCN, osteocalcin.

## 5 Discussion

Due to the rapid development of sequencing technologies and bioinformatics approaches in recent decades, various classes of ncRNAs have been discovered, including miRNAs, lncRNAs, and circRNAs. It is known that ncRNAs play an important biological role in the regulation of cellular mechanisms in both physiological and pathophysiological processes. NcRNAs can interact with and regulate various types of molecules through various molecular mechanisms, creating a complex regulatory network. NcRNA-mediated networks not only contain interactions between RNAs, but are also involved in genomics and proteomics in both the nucleus and cytoplasm.

Osteogenesis, angiogenesis and neurogenesis are complex processes involving several transcription factors and proteins. Moreover, a number of signaling cascades, particularly BMPs signal pathways regulate this process. Runx2 and osterix signaling cascades are other cascades that are involved in this processes. NcRNAs play an important role in the regulation of these transcription factors and signaling cascades in both health and disease. Experiments on various types of MSCs confirmed the importance of miRNAs, lncRNAs, and circRNAs in the regulation of the osteogenic process in these cells. These observations are of practical importance in medicine, especially in the treatment of bone diseases such as osteoporosis.

MiRNAs involved in oncogenesis mainly regulate the activity of Wnt, Notch, PI3Ks/AKT/mTOR, and BMPs signaling pathways. LncRNAs and circRNAs can directly influence the expression of these signaling pathways or oncogenic transcription factors. Moreover, they can serve as a molecular sponge for miRNAs. MALAT1/miR-494/BMP-2, MALAT1/miR142-3p/BMP-7, TUG1/miR-214/BMP-2, mm9_circ_009056/miR-22-3p/BMP-7, and hsa_circ_0005752/miR-496/BMP-2 are examples of such kind of interaction between miRNAs, lncRNAs and circRNAs by regulate BMPs signaling pathways in the context of biological processes and pathological conditions.

Briefly, several miRNAs, lncRNAs, and circRNAs have been found to alter the expression of BMPs signaling pathways, thus being involved in both normal physiological processes and oncogenesis or pathogenesis of bone disease.

## Funding

None.

## Author contributions

Conceptualization, Writing - original draft and Project administration: Ilgiz Gareev; Writing - review and editing, Investigation and Resources: Ural Mukhametov; Formal analysis and Methodology: Sergey Lyulin and Alina Shumadalova; Data curation: Sergey Lyulin, Dmitry Borzunov and Galina Sufianova; Validation and Visualization and Funding acquisition: Ural Mukhametov; Supervision: Sergey Lyulin. All authors have read and agreed to the published version of the manuscript.

## Declaration of competing interest

The authors declare no conflict of interest, financial or otherwise.
